# Patellar Tendon Reconstruction Using Autologous Hamstring Tendons for the Treatment of Extensive Patellar Tendon Ossification

**DOI:** 10.1111/os.13435

**Published:** 2022-08-24

**Authors:** Qian Liu, Dezhou Tang, Weihong Zhu, Yueming Chen

**Affiliations:** ^1^ Department of Orthopaedics, The Second Xiangya Hospital Central South University Changsha China

**Keywords:** Autograft, Hamstring tendon, Heterotopic ossification, Patellar fracture, Patellar tendon, Reconstruction

## Abstract

**Background:**

Extensive patellar tendon ossification is very uncommon and requires surgical intervention when ossification significantly affects knee function. While various approaches and grafts are available for reconstructing ruptured patellar tendons, there is a paucity of literature regarding the management of joint ankylosis due to severely ossified patellar tendons.

**Case Presentation:**

This is a case involving an extensively ossified patellar tendon after patellar and tibial tuberosity fracture fixation. Reconstruction of the patellar tendon was performed using ipsilateral semitendinosus and gracilis autografts. At the latest follow‐up of 12 months, the patient achieved knee flexion up to 120° with a slight extension lag and resumed daily activities.

**Conclusions:**

Autograft hamstring reconstruction of the patellar tendon is suitable for extensive heterotopic ossification of the patellar tendon, resulting in significant improvement in postoperative knee function. Similar patients may be referred for this reconstruction technique.

## The images provide a visual reference, including comparison of preoperative and postoperative patient related physical examination, imaging examination, basic intraoperative information and schematic presentation of surgical procedures. The relevant information of surgical technique and surgical effect can be seen intuitively.

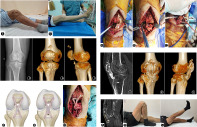

## Introduction

Patellar tendon ossification is rarely seen. Previous studies have reported patellar tendon ossification associated with antegrade or retrograde femoral nailing,^1^, ^2^ spinal cord injury,^3^ partial patellectomy,^4^ anterior cruciate ligament reconstruction (ACLR) using a bone‐patellar tendon‐bone graft (BPTB),^5^, ^6^ sleeve fractures of the patella or tibial tuberosity,^7^ and genetic disorder.^8^ Currently, there is no consensus regarding the standard treatment for patellar tendon ossification, especially when extensive ossification occurs that may require surgical intervention. We herein present a case of severe patellar tendon ossification treated with patellar tendon reconstruction utilizing ipsilateral semitendinosus and gracilis autografts.

## Case Report

**Fig. 1:**
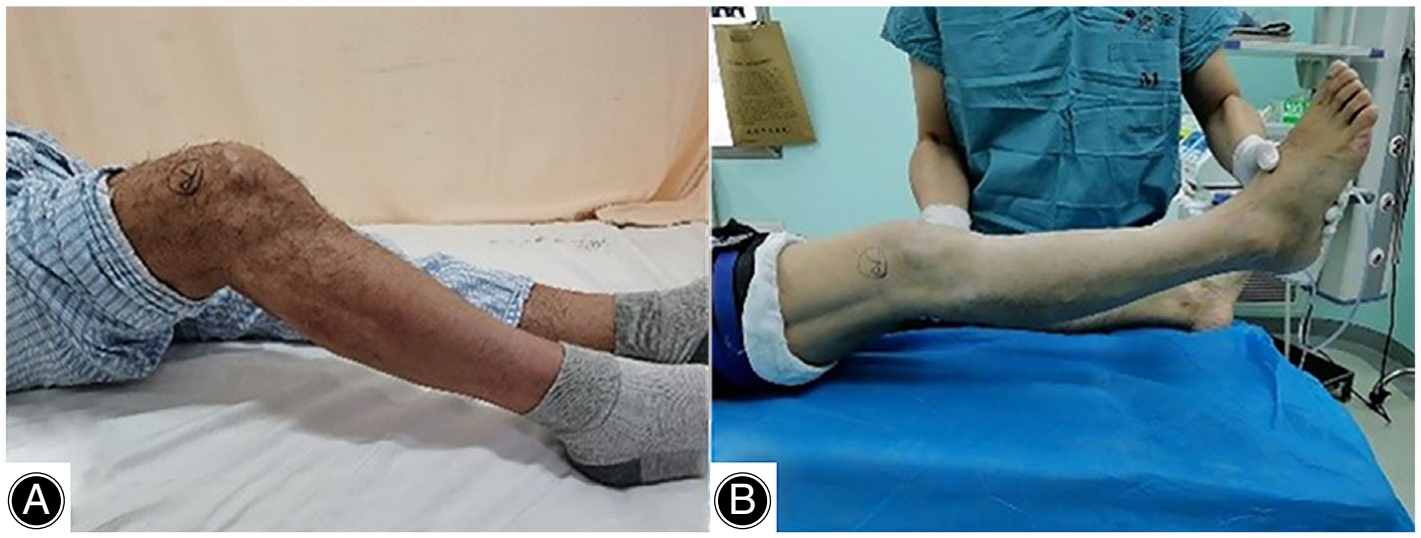
Preoperative knee range of motion: knee flexion (A) and extension (B).

**Fig. 2:**
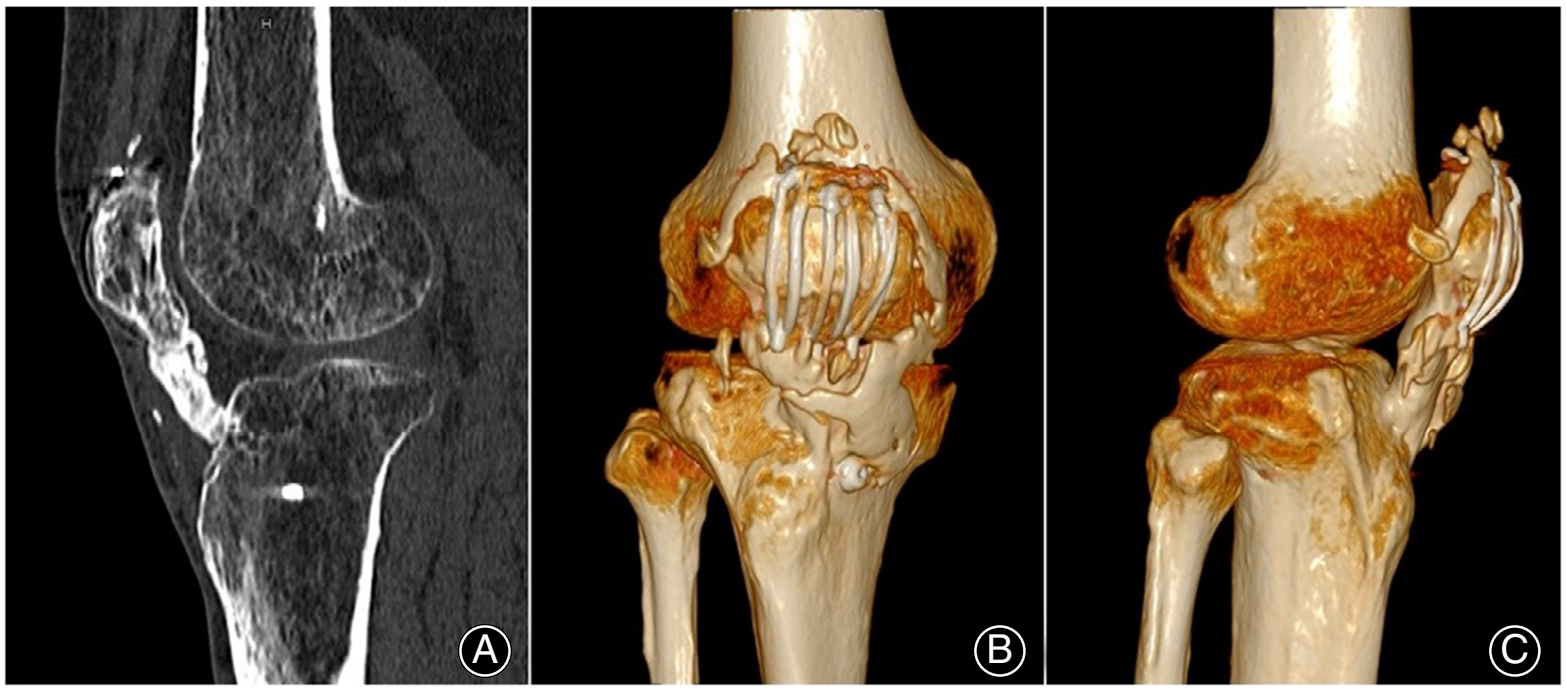
Sagittal CT scan image (A) and three‐dimensional reconstruction CT images (B, C) of the patient's right knee show extensive patellar tendon ossification throughout its whole length.

A 61‐year‐old male was presented to our hospital with progressive joint stiffness of his right knee. He underwent tension band wiring using K‐wires for a transverse patellar fracture and screw fixation for a tibial tuberosity fracture 2 years previously. One year of post‐surgery, the patient experienced a gradual loss of knee range of motion (ROM) and felt progressive hardening of the patellar tendon. He did not suffer any other injuries to the right knee. On physical examination, there was no knee swelling or tenderness. A hard and bony patellar tendon extending from the inferior pole of the patella to the tibial tuberosity was palpated. The knee ROM was limited to 15°–45° (Fig. Fig. 1A,B). No signs of joint instability or skin abnormity were noticed, and the HSS and Lysholm scores of the knee were 48 and 53, respectively. Radiography and computed tomography (CT) scanning revealed extensive ossification of the patellar tendon fused with the inferior pole of the patella and the tibial tuberosity (Fig. 2). Small ossification was present around the patella and within the quadriceps tendon. The height of the patella was normal. Blood tests covering lipids, glucose, electrolytes, and liver and renal function were conducted to rule out metabolic diseases, and the results were within normal ranges.

**Fig. 3:**
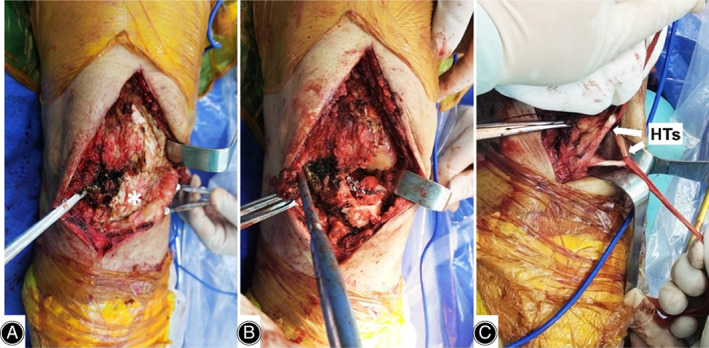
Intraoperative expose of the ossified patellar tendon (indicated by asterisk) (A), the removement of ossified tissue (B) and the harvesting of autologous hamstring tendons (C). HTs, hamstring tendons.

**Fig. 4:**
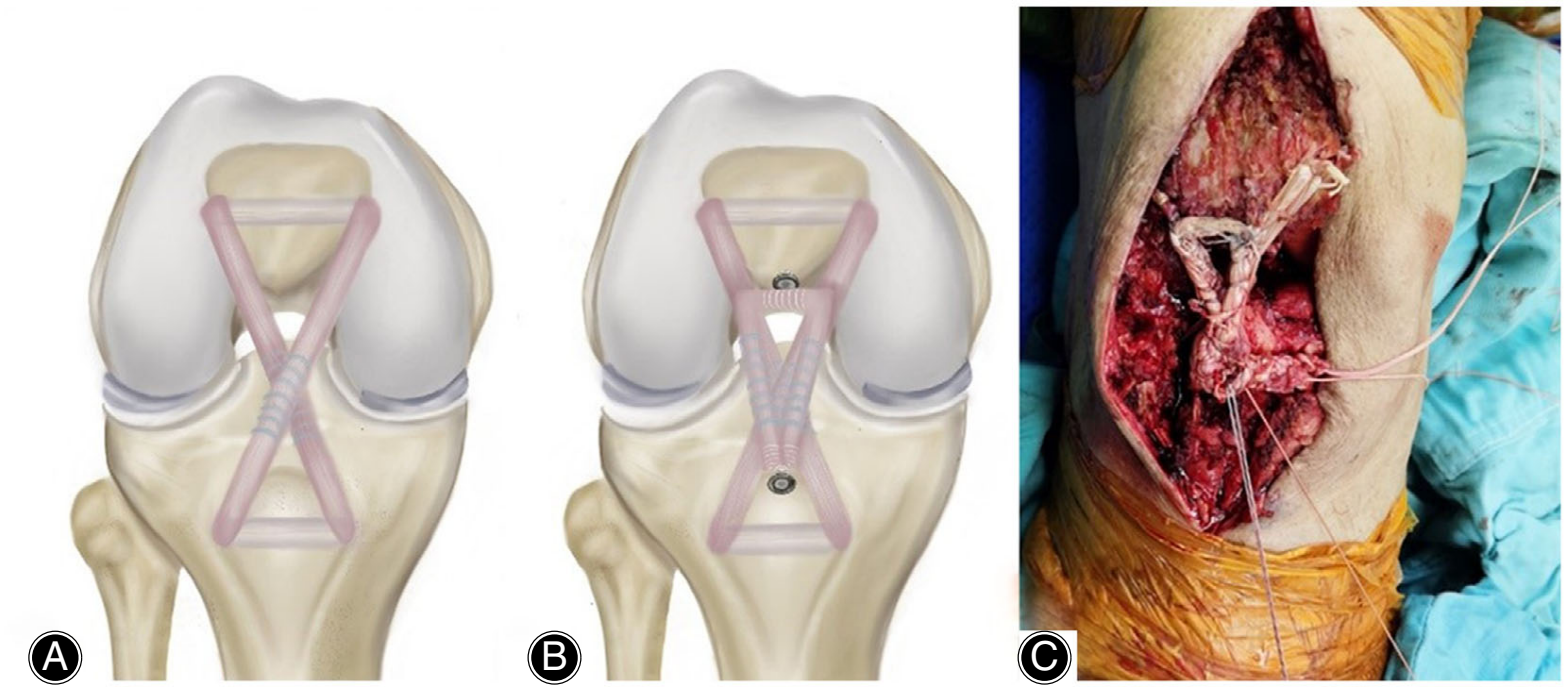
(A) The autologous semitendinosus tendon is passed through 4.5 mm transverse tibial and patellar tunnels in a criss crossed configuration. (B) The gracilis tendon is positioned in an inverted U‐shaped pattern and fixed to the patella and tibial tuberosity using two suture anchors. The gracilis tendon is sutured together with the underlying semitendinosus tendon. (C) Intraoperative photograph of the final completed patellar tendon reconstruction.

**Fig. 5:**
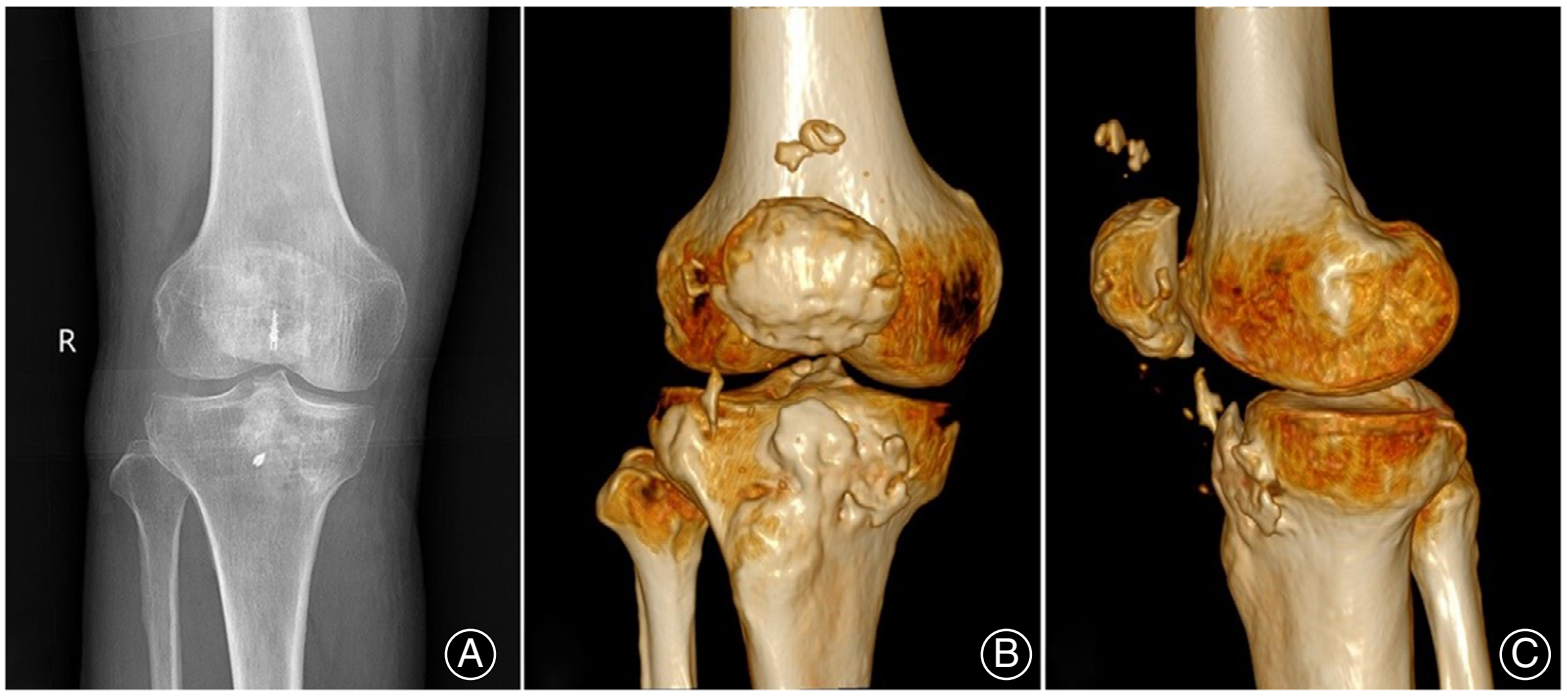
Postoperative anteroposterior radiograph (A) and three‐dimensional reconstruction CT images (B, C) revealing nearly complete debridement of the ossified patellar tendon. Note the suture anchors placed in the patella and tibia for the reconstruction of patellar tendon.

Surgical reconstruction of the patellar tendon was performed. Under general anesthesia, the patient was positioned supine, and the original incision was used to expose the patellar tendon. After removing the internal fixation devices, the ossified lesions were carefully excised along with surrounding scar tissue while preserving the intact tendon sheath to retain blood supply. The semitendinosus and gracilis tendons were then harvested using a tendon stripper (Fig. 3). Both ends of the tendon were whip stitched with #1 Vicryl sutures. A transverse tunnel was created by drilling with a 4.5‐mm bit over a guiding Kirschner pin in the lower half of the patella. Another transverse tibial tunnel was drilled approximately 1 cm posterior to the tibial tuberosity in the same manner. Subsequently, the semitendinosus tendon was passed through the two tunnels using a figure‐eight‐shaped PDS suture. The free ends of the tendon were sutured together at 30° of knee flexion with #2 Ultrabraid sutures (Smith & Nephew Inc., Andover, MA, USA). Next, the gracilis tendon was placed in an inverted U‐shaped pattern and fixed at the inferior pole of the patella and the tibial tuberosity with a 3.5‐mm suture anchor (TwinfixTi; Smith & Nephew Inc., Andover, MA, USA). In addition, the overlapping part of the semitendinosus and gracilis tendons was secured using #2 Ultrabraid sutures (Smith & Nephew Inc., Andover, MA, USA) to strengthen the construct (Fig. 4). Cerclage wiring was not used, as it requires removal and may cause fractures. A thorough cleaning was performed before layer‐by‐layer wound closure. The knee joint was easily flexed to 90° without tension intraoperatively. Postoperative radiography and CT demonstrated a normal patellar height and almost complete removal of the lesions (Fig. 5). Celecoxib was administered orally at 200 mg twice a day for 2 weeks without causing gastrointestinal distress.

**Fig. 6:**
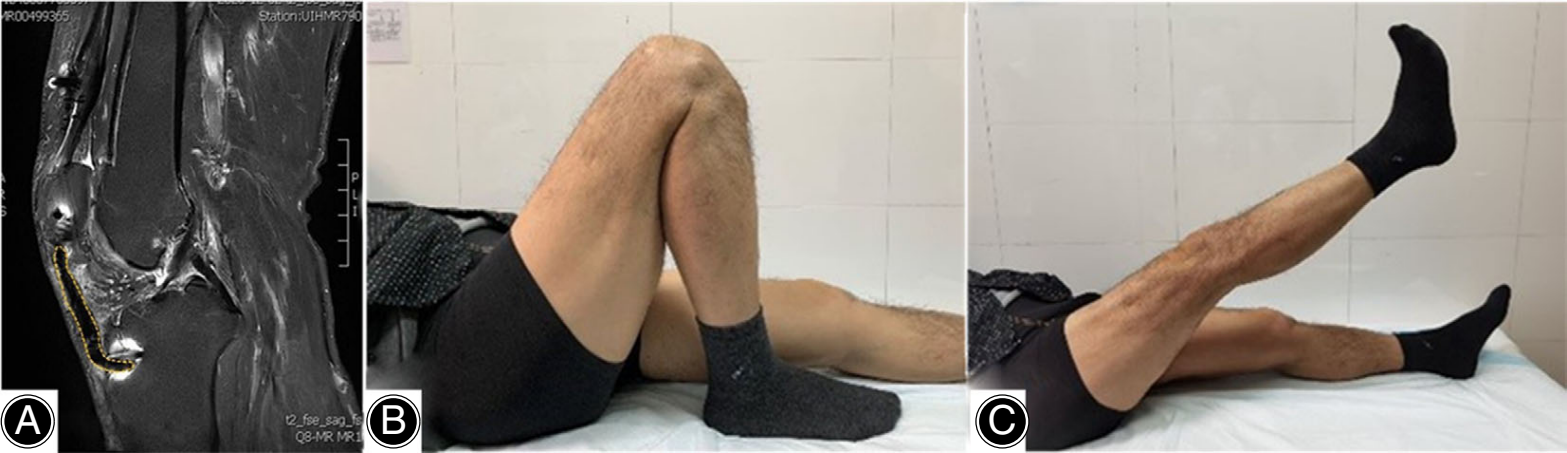
At 12 months follow‐up: (A) MRI of the right knee showing patellar tendon‐like structure on the sagittal plane (yellow dashed line); Postoperative range of motion of knee joint: knee flexion (B) and knee extension (C).

The patient was allowed to perform weight‐bearing as tolerated with the protection of a knee brace 1 day after the surgery. An ankle pump exercise was started immediately. Passive ROM was initiated as soon as possible and gradually increased to 90° in the first 4 weeks. At 6 weeks, active ROM was initiated. At the latest follow‐up (12 months), magnetic resonance imaging (MRI) showed a patellar tendon‐like structure on the sagittal plane, indicating successful reconstruction with our technique (Figure 6A). The right knee can flex up to 120° with a 5° extension lag (Fig. 6B,C). The HSS and Lysholm scores of the knee were 95 and 90, respectively. The patient had no pain and resumed daily activities.

## Discussion

Heterotopic ossification (HO) refers to the presence of lamellar bone within soft tissues. While the occurrence of HO is generally related to injury or inherited disease, its exact pathogenesis has not been completely elucidated. In clinical practice, HO resulting from trauma, surgery or neurogenic injury is most common (Table 1). The prevalence of HO has been demonstrated to range from 10% to 53% after central nervous system injury and approximately 40% after hip arthroplasty or acetabular fracture fixation.^9^, ^10^ Regarding HO of the patellar tendon, Bruijn *et al*.^7^ reported two children with sleeve fractures of the patella and tibial tuberosity who underwent conservative treatment. They suggested that early diagnosis and surgical intervention are essential to prevent HO. Yamamoto *et al*.^3^ presented a patient with extensive bilateral patellar tendon ossification and speculated that the cause of bilateral HO was the combination of traumatic and neurogenic injury. In addition to injury‐induced ossification, previous studies have shown HO of the patellar tendon after several surgical procedures, including ACLR with BPTB,^5^, ^6^ intramedullary nailing for femoral or tibial shaft fractures,^1^, ^2^ and partial or total patellectomy.^4^, ^11^ It is believed that insufficient cleaning of bone debris during surgery could contribute to the development of ectopic ossification in the patellar tendon.^2^, ^5^ For our patient who underwent operative fracture reduction and internal fixation, if thorough intraoperative rinsing had not been performed at the index surgery, it is possible that bone marrow or bone debris carrying osteogenic precursor cells would have been deposited on the patellar tendon. Through interplay with postoperative local inflammation and certain osteoinductive factors, such as bone morphogenetic proteins (BMPs), mesenchymal stem cells are recruited and differentiate toward the osteogenic lineage, eventually leading to the formation of HO.^9^, ^12^ Additionally, nonsteroidal anti‐inflammatory drug (NSAID) therapy has been widely acknowledged as an effective method of HO prophylaxis.^9^ Our patient was administered imrecoxib, a selective cyclooxygenase (COX)‐2 inhibitor, after the index surgery. However, he took it for only a few days due to gastrointestinal discomfort. The short administration of NSAIDs may also increase the risk of patellar tendon HO development.Compared with NSAID therapy, radiation has shown equal effectiveness in preventing HO after hip surgeries.^13^, ^14^ However, radiation is associated with the potential side effect of soft‐tissue contracture.^15^ Therefore, for patients who have limited joint ROM, as in our case, the utility of NSAIDs for prophylaxis against HO is recommended over radiotherapy postoperatively.

**TABLE 1:** Causes and management of patellar tendon ossification reported in literature

Authors	Age(years)	Sex	Causes	Treatment	Methods
Tan *et al*.^2^	25	Man	After intramedullary nail fixation of femoral shaft fractures, the continuous movement of the knee joint was limited for 2 years	Conservative treatment	The patient refused treatment
Yamamoto *et al*.^3^	84	Man	Trauma and neurogenic injury lead to extensive bilateral patellar calcification	Conservative treatment	No knee surgery was performed because ossification caused little dysfunction
Camillieri *et al*.^5^	42	Man	Anterior knee pain and gradual thickening and hardening of the patellar tendon after the reconstruction of the anterior cruciate ligament using bonepatellar tendon technique	Surgical treatment Remove the ossified structures around the patellar tendon	
Erdil *et al*.^6^	36	Man	Heterotopic patellar tendon ossification occurred after anterior cruciate ligament reconstruction with autogenous bone‐patellar tendon‐bone graft	Surgical treatment	Surgical excision of the ossified part
Bruijn *et al*.^7^	12	Girl	Heterotopic ossification of the patella and patellar tendon occurred after conservative treatment of sleeve fractures of the patella and tibial trochanter in two children	Surgical treatment	The ossified structure was surgically removed and the patella tendon and knee support band were reconstructed and reinforced with wire

Although various surgical techniques and grafts have been utilized to reconstruct ruptured patellar tendons, there is a paucity of literature describing reconstruction for extensively ossified patellar tendons. In this case, the patient had almost no remnant tissue after debridement, as ossification involves the entire patellar tendon. Therefore, it was quite challenging to restore the extensor mechanism and improve his quality of life. We first passed a semitendinosus autograft through two transosseous tunnels in the patellar and tibial tuberosities to achieve a crossed configuration. Then, a gracilis autograft was fixed in an inverted U‐shape on the patella and tibial tuberosity with suture anchors for augmentation. Finally, the overlapping portion of these two autografts was sutured together to obtain a strong construct. Similar to our technique, Leo *et al*.^16^ harvested autograft hamstring tendons and passed them through a tibial transverse tunnel. The semitendinosus and gracilis tendons were delivered proximally in a crossed and U‐shaped pattern, respectively. The ends of both tendons were inserted into the inferior patellar sockets and secured with interference screws. Their method may serve as an alternative when the patient's hamstring tendon does not have sufficient length to pass through a transverse patellar tunnel. It has been shown that a variety of autogenous grafts have been used for the reconstruction of patellar tendons, among which hamstring autografts are the most popular choice.^17^ The harvesting and handling of hamstring tendons are relatively easy, and they can ensure the reestablishment of a stable and strong extensor mechanism. Furthermore, the preserved tendon sheath may play an important role in promoting neovascularization after patellar tendon reconstruction and, thus, facilitating tendon remodeling and healing.

## Conclusions

Extensive patellar tendon ossification is very rare, and surgical intervention is needed when it impairs knee function. This article presented an unusual case involving a severely ossified patellar tendon and reconstruction of the patellar tendon using ipsilateral hamstring tendons. The surgical approach yielded favorable results and could be considered for such challenging patients. Future studies with longer follow‐ups are warranted to validate its efficacy.

## Author Contribution

Liu Qian and Tang Dezhou, co‐first authors of this paper, were responsible for information collection and manuscript writing. Yueming Chen participated in the surgery and the patient's treatment. Weihong Zhu was the lead surgeon and the corresponding author of this paper.
